# Efficacy of prognostic nutrition index in combination with D-dimer in predicting postoperative clinical adverse events after acute type A aortic dissection: a single center retrospective study

**DOI:** 10.3389/fcvm.2023.1210725

**Published:** 2023-10-09

**Authors:** Linfeng Xie, Jian He, Xinfan Lin, Zhaofeng Zhang, Xinghui Zhuang, Debin Jiang

**Affiliations:** ^1^ Department of Cardiovascular Surgery, Fujian Medical University Union Hospital, Fuzhou, China; ^2^ Key Laboratory of Cardio-Thoracic Surgery (Fujian Medical University), Fujian Province University, Fuzhou, China; ^3^ Fujian Provincial Center for Cardiovascular Medicine, Fuzhou, China

**Keywords:** acute type A aortic dissection, prognostic nutrition index, D-dimer, clinical adverse events, predictive efficacy

## Abstract

**Background:**

The aim of this study was to identify the predictive factors for adverse clinical events after surgery in patients with acute type A aortic dissection (AAAD), and to explore the predictive value of preoperative prognostic nutritional index (PNI) combined with D-dimer for these events.

**Methods:**

This study was a retrospective analysis of clinical data of 153 patients with AAAD who underwent emergency surgery at our center from January 2019 to January 2022. Patients were divided into adverse event group and non-adverse event group based on whether they experienced adverse clinical events after surgery. Univariate and multivariable logistic regression analyses were performed to identify the risk factors for adverse events, and the predictive efficacy was evaluated by the area under the receiver operating characteristic curve (ROC-AUC).

**Results:**

A total of 153 AAAD patients were included in the study, and were divided into the adverse event group (*n* = 46) and the non-adverse events group (*n* = 107) based on whether or not they experienced clinical adverse events after surgery. The optimal cutoff value was determined using ROC curves, and multivariate logistic regression analysis was performed. Ultimately, it was found that preoperative PNI < 42.45 and D-dimer > 15.05 were independent predictors of postoperative clinical adverse events in AAAD patients. The odd ratios (OR) value for preoperative PNI < 42.45 is 3.596 [95% Confidence Interval (CI): 1.508–8.923, *p *= 0.004], while the OR value for D-dimer > 15.05 is 7.572 [95% CI: 3.094–20.220, *p *< 0.001]. The combination of these two indicators has a high predictive value (AUC = 0.843, 95% CI: 0.774–0.912, *p *< 0.001) and is superior to using either variable alone.

**Conclusion:**

Preoperative PNI < 42.45 and D-dimer > 15.05 are independent predictive factors for postoperative adverse events during hospitalization in patients with AAAD. The combination of these two indicators can improve the predictive accuracy, which is superior to using either variable alone.

## Introduction

Acute aortic dissection is a highly lethal and high-risk cardiovascular emergency. Among them, acute type A aortic dissection (AAAD) is the most dangerous situation (1–3). The previous research results showed that the overall incidence rate of AAAD is approximately 4.7 per 100,000 population, but there are significant regional variations in the incidence rates (4). A study by Meszaros et al. indicated that the average age of onset of AAAD is around 50 years old (5). Its clinical features include an acute onset, rapid progression, and high mortality rate. Some studies have reported that the in-hospital mortality rate associated with AAAD is approximately 22% (6), and without surgical treatment, it is as high as 82% at 1 year (7). The only effective treatment for AAAD is emergency surgical repair. Although surgical techniques and perioperative management have made great progress compared to the past, the prognosis of AAAD after surgery is significantly worse compared to conventional cardiovascular surgery due to the characteristics of the disease and the complexity of the surgery (7–9). Therefore, it is necessary to identify sensitive preoperative prognostic indicators to predict the prognosis of AAAD patients, which can effectively improve perioperative treatment measures and help clinical doctors better evaluate the early outcomes after AAAD surgery.

Inflammatory responses and changes in coagulation function are integral to the development of aortic dissection and are closely related to its pathogenesis and prognosis (10, 11). Previous studies have shown that some inflammation-related biomarkers, such as C-reactive protein (CRP) and D-dimer, are considered to be associated with an adverse prognosis after AAAD surgery (12–14). The prognostic nutritional index (PNI) is a new systemic inflammation marker calculated based on serum albumin levels and peripheral lymphocyte count. It was originally widely used to evaluate the long-term outcomes and prognosis of gastrointestinal cancer patients (15, 16). In recent years, PNI has been shown to be associated with the prognosis of heart failure and coronary heart disease patients (17–19). Studies have found that a low PNI during the perioperative period is a risk factor for in-hospital mortality in AAAD patients (20, 21). However, as a single indicator, the accuracy of using PNI to predict postoperative outcomes after AAAD surgery is not sufficient. Therefore, it is necessary to combine other indicators for a systematic evaluation. Previous studies have shown that the coagulation function is closely associated with the prognosis of AAAD. D-dimer, as a major indicator reflecting the coagulation status of the body, can serve as a key biomarker for predicting postoperative outcomes (22). Through univariate and multivariate regression analysis, we found that PNI and D-dimer are independent risk factors for clinical adverse events after AAAD surgery. Therefore, we speculate that the composite index formed by PNI combined with D-dimer can provide effective clinical predictive information for clinical adverse events after AAAD surgery. The aim of this study is to explore the application of two indicators combined in predicting the risk of adverse clinical events after AAAD surgery.

## Materials and methods

### Study design and setting

A single center retrospective study was used to investigate the clinical data of AAAD patients admitted to our center. These patients were admitted to hospital for emergency surgery from January 2019 to January 2022. Since this is a retrospective study and there is no need to obtain informed consent, this study was approved by the Ethics Committee of the affiliated Union Hospital of Fujian Medical University, which is in line with the Helsinki Declaration.

The inclusion criteria of this study were: AAAD diagnosed by computed tomography thoracic aortography or magnetic resonance imaging; over 18 years of age; emergency surgical treatment after admission. Patients with the following conditions are excluded: patients whose time from onset to hospitalization is more than 48 h; patients with long-term use of drugs that affect blood cell count; patients with malignant tumors, autoimmune diseases, severe infectious diseases and chronic organ dysfunction. The serological samples of all patients were drawn from venous blood without medication before emergency operation.

By measuring the level of D-dimer, serum albumin and lymphocyte count, the formula (10 × serum albumin (g/dL) + 0.005 × lymphocyte count (per mm^3^)) was used to calculate PNI (16), and the relationship between PNI, D-dimer and clinical adverse events after operation was analyzed.

## Definition of clinical adverse events

Clavien–Dindo grading is a general surgical complication grading system, which can also be used to grade the severity of complications after cardiovascular surgery (23). The postoperative clinical adverse events in this study were defined as complications of Clavien–Dindo grade III or above, including single or multiple organ dysfunction and postoperative death (24). Single organ dysfunction includes renal insufficiency requiring dialysis treatment, cardiac dysfunction requiring left ventricular assist device or intra-aortic balloon pump (IABP) therapy, neurological deficits requiring reintubation, tracheostomy or radiological and neurosurgical interventions, irreversible spinal cord injury, and intestinal ischemia requiring surgical intervention. Multiple organ dysfunction is defined as simultaneous or sequential dysfunction of two or more organs or systems caused by various clinical factors (25).

## Data collection

We collected clinical data of each patient from the hospital's medical record system and observed and summarized various indicators before and during surgery. Preoperative indicators include: (1) demographic data: gender, age, body mass index (BMI); (2) past medical history: smoking history, drinking history; (3) comorbidities: hypertension, diabetes, coronary artery disease, history of cerebrovascular disease, chronic obstructive pulmonary disease, Marfan syndrome, hepatic dysfunction and renal insufficiency; (4) preoperative general condition: aortic valve regurgitation (moderate or above), pericardial effusion (moderate or above), left ventricular ejection fraction (LVEF). Preoperative laboratory tests: red blood cell count, white blood cell count, leukomonocyte, hemoglobin, platelet, albumin, alanine aminotransferase, aspartate aminotransferase, serum creatinine, D-dimer, prothrombin time (PT), B-type natriuretic peptide, C-reactive protein, troponin-I. Intraoperative indicators include: processing method of the aortic root, total operation time, cardiopulmonary bypass time, aortic cross-clamp time, cerebral perfusion time time, deep hypothermic circulatory arrest time and intraoperative blood product input (red blood cells, plasma, platelets). The postoperative complications were classified into two groups based on whether or not Clavien–Dindo grade III or higher surgical complications occurred: the no-adverse events group and the adverse events group.

## Surgical technique

The surgery was performed under general anesthesia and cardiopulmonary bypass support. The specific surgical procedure was described in detail in Chen et al.'s previous study, including reconstruction of the aortic root, replacement of the ascending aorta, and implantation of a modified triple-branch stent graft (26, 27).

## Statistical analysis

All statistical analyses were performed using SPSS 23.0 and R software (4.2.2). Continuous variables were expressed as mean ± standard deviation or interquartile range, while categorical variables were expressed as frequency, ratio, and percentage. The Kolmogorov–Smirnov test was used to check the normality of the distribution of continuous variables. Student t-tests were used for intergroup comparison of continuous variables that followed a normal distribution, while Mann–Whitney U tests were used for those that did not follow a normal distribution. The chi-square test or Fisher's exact test was used for categorical variables. Predictive variables with *p *< 0.05 in the univariate analysis were included in a multivariate logistic regression analysis to identify independent risk factors for postoperative adverse events. Receiver operating characteristic (ROC) curves were constructed to determine the optimal cutoff values for PNI, D-dimer, and combination variables in predicting postoperative adverse events, and the area under the curve (AUC) was calculated. The predictive performance of the combined indicators will be evaluated using AUC, net reclassification improvement (NRI), and integrated discrimination improvement index (IDI). A difference was considered statistically significant when *p *< 0.05.

## Results

**Figure 1:**
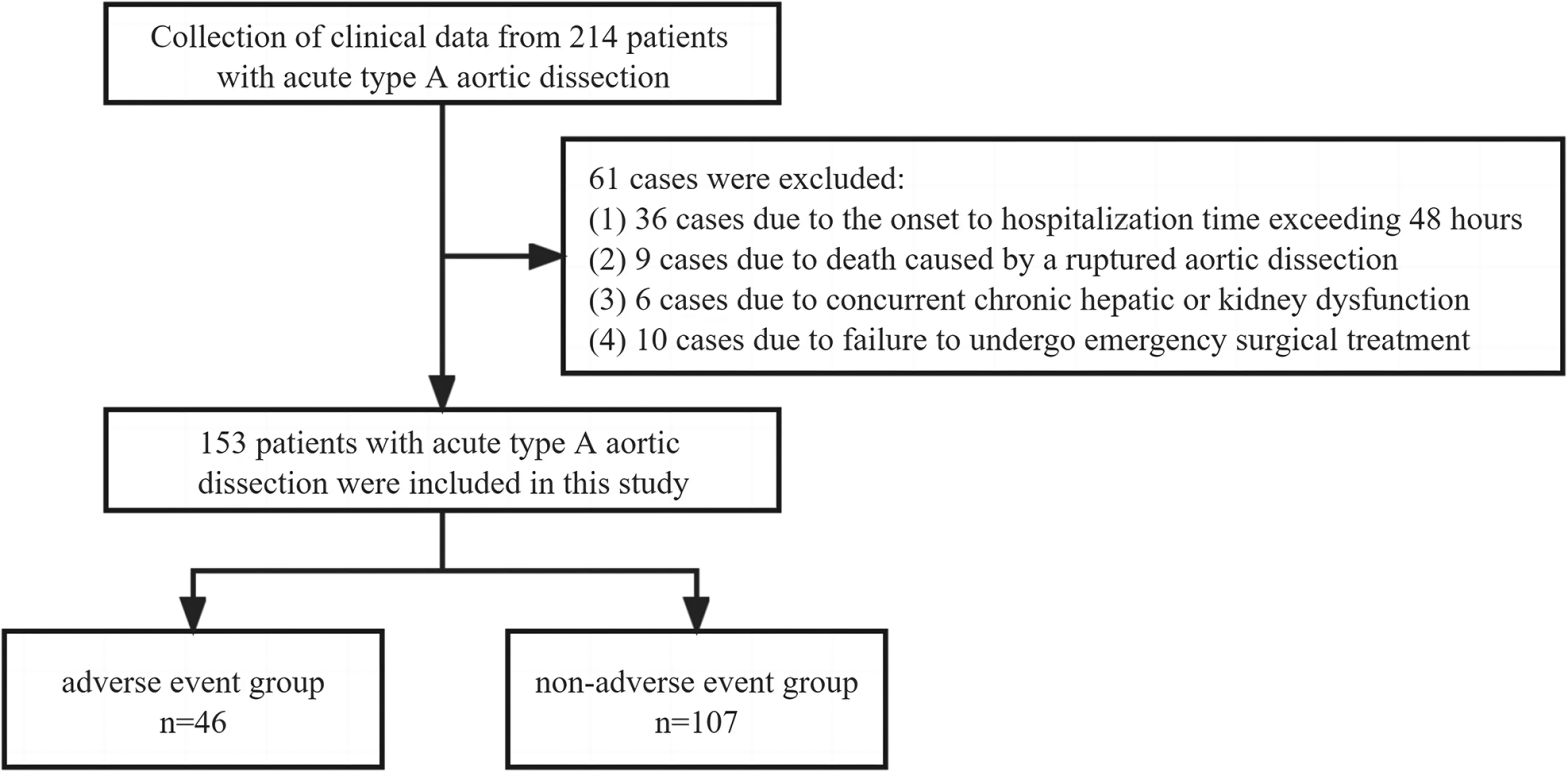
Grouping process and exclusion criteria for AAAD patients.

This study diagnosed a total of 214 patients with AAAD between January 2019 and January 2022. Among them, 36 cases were excluded due to the onset-to-hospitalization time exceeding 48 h, 9 cases were excluded due to death caused by a ruptured aortic dissection, 6 cases were excluded due to concurrent chronic hepatic or kidney dysfunction, and 10 cases were excluded due to failure to undergo emergency surgical treatment. In the end, a total of 153 patients were included in this study (Figure 1).

The baseline data comparison of the patients showed that the two groups of patients had similar baseline data such as gender, age, and body mass index (BMI), but the preoperative PNI of the adverse event group was significantly lower than that of the non-adverse event group (39.60 ± 5.68 vs. 44.55 ± 4.63, *p *< 0.001). Laboratory examination results showed that the leukomonocyte [0.71 (0.56, 1.02) vs. 1.01 (0.83, 1.37)], albumin (35.49 ± 5.13 vs. 39.00 ± 3.84), and platelet [151.00 (128.00, 196.00) vs. 176.00 (145.00, 206.00)] of the adverse event group were significantly lower than those of the non-adverse event group, while D-dimer (17.40 ± 5.47 vs. 9.83 ± 7.28), AST [33.00 (22.00, 71.00) vs. 24.00 (19.00, 34.00)], PT [14.30 (13.50, 15.80) vs. 13.80 (13.10, 14.50)], and troponin-I [0.013 (0.004, 0.165) vs. 0.005 (0.002, 0.033)] were significantly higher than those of the non-adverse event group. The differences in the clinical indicators between the two groups were statistically significant (*p *< 0.05) (Tables 1, 2). The intraoperative comparison results showed that there were no significant differences in the total operation time, cardiopulmonary bypass (CPB) time, aortic cross-clamp (ACC) time, cerebral perfusion time, and deep hypothermic circulatory arrest (DHCA) time between the two groups of patients. The aortic root handling methods and intraoperative blood product transfusion were also similar between the two groups (Table 3). The occurrence of postoperative complications in the adverse event group is shown in Table 4.

**Table 1:** Comparison of preoperative condition between the two groups.

Valuables	Non-adverse event group (*n* = 107)	Adverse event group (*n* = 46)	*P* value
Gender (male), *n* (%)	84 (78.5%)	35 (76.1%)	0.742
Age (year), mean (±SD)	53.15 ± 12.46	55.09 ± 12.97	0.388
BMI (Kg/m^2^), median [IQR]	23.97 [21.27, 25.67]	23.26 [21.63, 25.10]	0.510
Smoking history, *n* (%)	54 (50.5%)	17 (37.0%)	0.124
Drinking history, *n* (%)	52 (48.6%)	20 (43.5%)	0.561
Hypertension, *n* (%)	64 (59.8%)	29 (63.0%)	0.707
Diabetes, *n* (%)	5 (4.7%)	1 (2.2%)	0.465
Coronary artery disease, *n* (%)	4 (3.7%)	2 (4.3%)	0.859
History of cerebrovascular disease, *n* (%)	7 (6.5%)	2 (4.3%)	0.597
Chronic obstructive pulmonary disease, *n* (%)	1 (0.9%)	2 (4.3%)	0.163
Marfan syndrome, *n* (%)	3 (2.8%)	2 (4.3%)	0.622
Hepatic dysfunction, *n* (%)	1 (0.9%)	2 (4.3%)	0.163
Renal insufficiency, *n* (%)	3 (2.8%)	3 (6.5%)	0.277
LVEF (%), median [IQR]	63.70 [60.70, 67.10]	63.50 [60.20, 67.80]	0.827
Pericardial effusion (medium or above), *n* (%)	19 (17.8%)	6 (13.0%)	0.470
Aortic valve regurgitation (medium or above), *n* (%)	32 (29.9%)	13 (28.3%)	0.838
Prognostic nutritional index, mean(±SD)	44.55 ± 4.60	39.60 ± 5.61	**<0**.**001**

SD, standard deviation; IQR, interquartile range; BMI, body mass index; LVEF, left ventricular ejection fractions.

Bold values indicate a *p*-value less than 0.05, indicating a statistically significant difference between groups.

**Table 2:** Comparison of preoperative laboratory examination between the two groups.

Valuables	Non-adverse event group (*n* = 107)	Adverse event group (*n* = 46)	*P* value
White blood cell count (×10^9^/L), mean (±SD)	12.47 ± 3.56	12.79 ± 3.85	0.622
Red blood cell count (×10^12^/L), mean(±SD)	4.36 ± 0.52	4.31 ± 0.65	0.578
Leukomonocyte (×10^9^/L), median [IQR]	1.01 [0.83, 1.37]	0.71 [0.56, 1.02]	**<0**.**001**
Heamoglobin (g/L), mean (±SD)	132.28 ± 17.21	128.59 ± 17.29	0.226
Platelet (×10^9^/L), median [IQR]	176.00 [145.00, 206.00]	151.00 [128.00, 196.00]	**0**.**025**
Albumin (g/L), mean (±SD)	39.00 ± 3.84	35.49 ± 5.13	**<0**.**001**
ALT (IU/L), median [IQR]	20.00 [13.00, 31.00]	24.00 [15.00, 44.00]	0.084
AST (IU/L), median [IQR]	24.00 [19.00, 34.00]	33.00 [22.00, 71.00]	**0**.**005**
Serum creatinine (μmol/L), median [IQR]	89.00 [72.00, 114.00]	93.00 [67.00, 143.00]	0.474
D-dimer (μg/ml), mean (±SD)	9.83 ± 7.28	17.40 ± 5.47	**<0**.**001**
PT (s), median [IQR]	13.80 [13.10, 14.50]	14.30 [13.50, 15.80]	**0**.**040**
B-type natriuretic peptide (pg/ml), median [IQR]	224.00 [110.00, 678.00]	345.00 [156.00, 895.00]	0.123
Troponin-I (μg/L), median [IQR]	0.005 [0.002, 0.033]	0.013 [0.004, 0.165]	**0**.**004**
CRP (mg/L), median [IQR]	10.41 [3.51, 36.88]	8.46 [3.24, 25.63]	0.586

SD, standard deviation; IQR, interquartile range; PT, prothrombin time; ALT, alanine aminotransferase; AST, aspartate aminotransferase; CRP, c-reactive protein.

Bold values indicate a *p*-value less than 0.05, indicating a statistically significant difference between groups.

**Table 3:** Comparison of intraoperative conditions between the two groups.

Valuables	Non-adverse event group (*n* = 107)	Adverse event group (*n* = 46)	*P* value
Intraoperative time			
Operative time (min), median [IQR]	285.00 [255.00, 334.00]	300.00 [271.00, 330.00]	0.183
Cardiopulmonary bypass time (min), median [IQR]	139.00 [120.00, 160.00]	138.00 [128.00, 151.00]	0.794
Aortic cross-clamp time (min), median [IQR]	62.00 [53.00, 83.00]	62.00 [53.00, 81.00]	0.967
Cerebral perfusion time (min), median [IQR]	9.00 [5.00, 11.00]	7.00 [6.00, 10.00]	0.577
DHCA time (min), median [IQR]	4.00 [3.00, 5.00]	4.00 [3.00, 5.00]	0.186
Intraoperative blood transfusion			
Red blood cell transfusion volume (U), median [IQR]	4.00 [2.00, 4.00]	4.00 [2.00, 4.50]	0.831
Plasma transfusion volume (ml), median [IQR]	400.00 [200.00, 500.00]	400.00 [0, 600.00]	0.327
Platelet transfusion volume (U), median [IQR]	2.00 [0.80, 10.00]	1.00 [0, 10.00]	0.306
Aortic root concomitant procedure 0.759			0.759
No treatment, *n* (%)	48 (44.9%)	18 (39.1%)	
Sinus forming, *n* (%)	40 (37.4%)	20 (43.5%)	
Bentall, *n* (%)	19 (17.8%)	8 (17.4%)	

SD, standard deviation; IQR, interquartile range; DHCA, deep hypothermic circulatory arrest.

**Table 4:** In-hospital postoperative clinical adverse events in patients with AAAD.

Postoperative clinical adverse events (*n* = 153)	Number	Percentage
Renal failure (need CRRT)	27	17.65%
Respiratory failure	7	4.58%
Gastrointestinal bleeding	6	3.92%
Low cardiac output syndrome (need IABP)	2	1.31%
Ventricular fibrillation	5	3.27%
Permanent neurological deficits	14	9.15%
Sepsis	11	7.19%
Secondary thoracotomy	1	0.65%
Secondary intubation	7	4.58%
Tracheotomy	3	1.96%
Pericardial effusion	6	3.92%
Myocardial ischemia	2	1.31%
Death	12	7.84%

AAAD: acute type A aortic dissection; CRRT: continuous renal replacement therapy; IABP: intra-aortic balloon pump.

**Figure 2:**
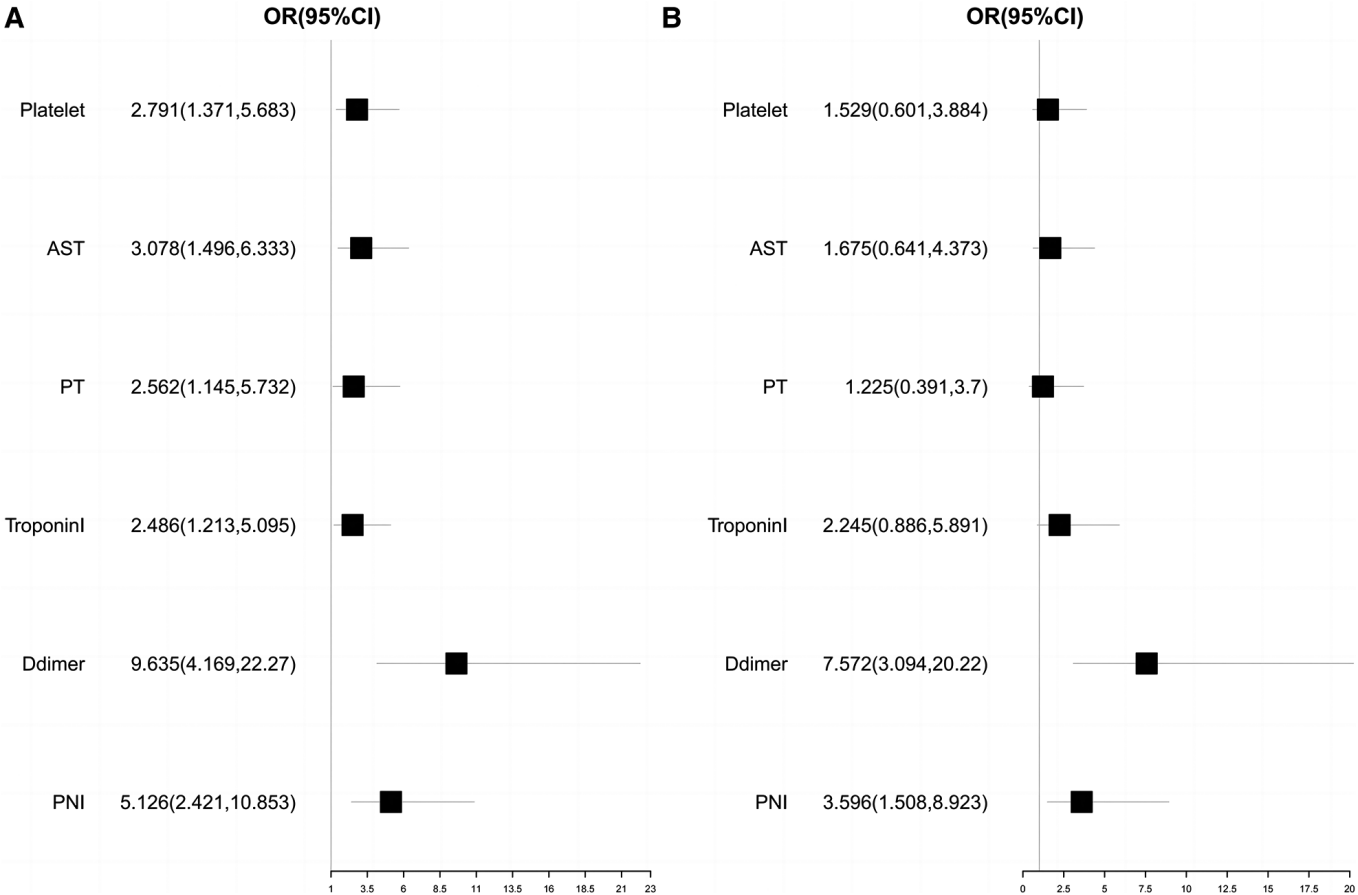
Univariate and multivariate logistics regression analysis forest plot. (**A**) Univariate logistics regression analysis forest plot; (**B**) Multivariate logistics regression analysis forest plot.

The optimal cut-off values of the clinical characteristic variables for which there were statistically significant differences between groups were calculated by drawing a ROC curve. Subsequently, univariate and multivariate logistic regression analyses were performed, and the results are presented in Table 5. A forest plot depicting the odds ratio (OR) and their 95% confidence interval (CI) was generated based on the results, as shown in Figure 2.

**Table 5:** Univariate and multivariate analysis of postoperative clinical adverse events.

Valuables	Univariate analysis			Multivariate analysis		
	OR	95% CI	*P* value	OR	95% CI	*P* value
PNI < 42.45	5.126	[2.421, 10.853]	**<0**.**001**	3.596	[1.508, 8.923]	**0**.**004**
Platelet < 154 (×10^9^/L)	2.791	[1.371, 5.683]	**0**.**005**	1.529	[0.601, 3.884]	0.369
AST > 32 (IU/L)	3.078	[1.496, 6.333]	**0**.**002**	1.675	[0.641, 4.373]	0.289
D-dimer > 15.05 (μg/ml)	9.635	[4.169, 22.270]	**<0**.**001**	7.572	[3.094, 20.220]	**<0**.**001**
PT > 15.3(s)	2.562	[1.145, 5.732]	**0**.**022**	1.225	[0.391, 3.700]	0.721
Troponin-I > 0.007 (μg/L)	2.486	[1.213, 5.095]	**0**.**013**	2.245	[0.886, 5.891]	0.092

PNI, prognostic nutritional index; PT, prothrombin time; AST, aspartate aminotransferase.

Bold values indicate a *p*-value less than 0.05, indicating a statistically significant difference between groups.

We found that when the optimal cutoff value was 42.45, the sensitivity of preoperative PNI was 0.692, specificity was 0.696, and the AUC value was 0.752; the optimal cutoff value for D-dimer was 15.05, with a sensitivity of 0.826, specificity of 0.701, and an AUC value of 0.770. The results of the multivariate logistic regression analysis showed that a PNI < 42.45 and a D-dimer > 15.05 μg/ml were independent risk factors for postoperative clinical adverse events in AAAD patients.

**Figure 3:**
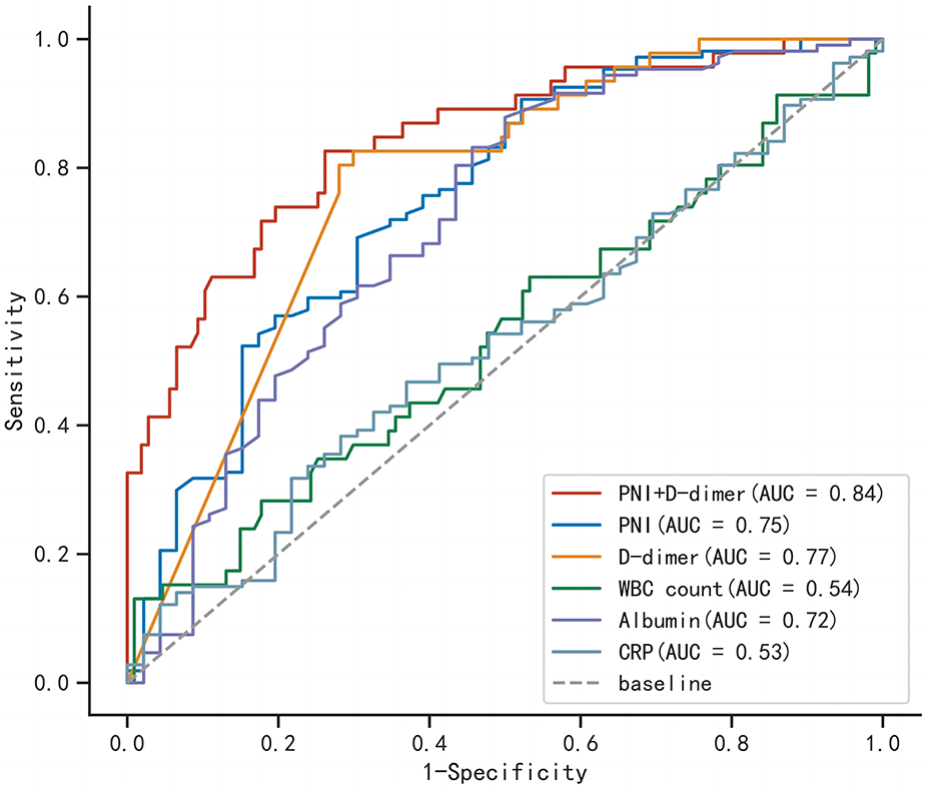
Comparison of receiver operating characteristic curves for combined indicators and single indicators.

In order to better predict postoperative clinical adverse events, we combined the above clinical indicators (preoperative PNI combined D-dimer) and calculated the AUC of the combined index to be 0.843 (95% CI was 0.774–0.912, *p *< 0.001), with a sensitivity of 0.826 and specificity of 0.738. The AUC value of the combined index was more predictive than using a single indicator alone (Table 6). We also assessed the effectiveness and accuracy of the combined indicators in clinical prediction using the NRI and IDI. The results, shown in Table 7, indicate that the combined indicators have higher predictive accuracy compared to traditional individual predictors such as PNI or D-dimer. In order to compare the predictive performance of the combined index with traditional single biochemical indicators, we drew ROC curves to compare the combined index (preoperative PNI combined D-dimer) with commonly used clinical indicators of inflammation severity (WBC, CRP) and nutritional status (ALB). The results are shown in Figure 3, and the combined index still outperforms the single biochemical indicators in predicting outcomes.

**Table 6:** Predictive value of preoperative PNI combined D-dimer for postoperative clinical adverse events.

Valuables	AUC	Cut-off value	95% CI	*P* value
PNI	0.752	42.45	[0.665, 0.839]	**<0**.**001**
D-dimer	0.770	15.05	[0.694, 0.847]	**<0**.**001**
PNI combined D-dimer	0.843	/	[0.774, 0.912]	**<0**.**001**

PNI, prognostic nutritional index.

Bold values indicate a *p*-value less than 0.05, indicating a statistically significant difference between groups.

**Table 7:** Comparison of the predictive performance of combined indicators vs. single indicators.

Valuables	NRI [95% CI]	*P* value	IDI [95% CI]	*P* value
PNI combined D-dimer vs. PNI	0.354 [0.157, 0.552]	**<0**.**001**	0.142 [0.089, 0.195]	**<0**.**001**
PNI combined D-dimer vs. D-dimer	0.348 [0.182, 0.515]	**<0**.**001**	0.115 [0.055, 0.175]	**<0**.**001**

NRI, net reclassification index; IDI, integrated discrimination improvement index; PNI, prognostic nutritional index.

Bold values indicate a *p*-value less than 0.05, indicating a statistically significant difference between groups.

In summary, preoperative PNI and D-dimer are effective predictive indicators for adverse events after AAAD surgery, and their combined use is more accurate.

## Discussion

AAAD is an extremely dangerous cardiovascular emergency. If damaged aorta is not repaired by surgery in a timely manner, the mortality rate within 48 h is about 50% (28). Despite emergency surgery being performed, the mortality rate remains high due to numerous postoperative complications. Therefore, early risk prediction of clinical adverse events and taking timely and effective measures for treatment can greatly help reduce in-hospital mortality. It is crucial to identify predictive factors that can forecast the risk of clinical adverse events after AAAD surgery. Currently, many studies have shown that certain blood biochemical indicators may have important significance in predicting the postoperative prognosis of AAAD, such as inflammatory factors and coagulation function indicators (29, 30). However, the clinical accuracy of predicting with a single indicator still needs to be improved. Therefore, this study aims to explore the clinical application value of combining multiple indicators to predict clinical adverse events after AAAD surgery.

Previous studies have shown that local and systemic inflammatory responses play a crucial role in the development of AAAD (31–33). Inflammatory factors such as interleukin-6, procalcitonin, CRP are significantly elevated in the serum of most AAAD patients, and the degree of inflammatory response is often closely related to the prognosis (34, 35). As a composite index of systemic inflammation, PNI takes into account the degree of current inflammatory response and corresponding nutritional status, and the immune and nutritional status is closely related to the progression and prognosis of cardiovascular disease (36, 37). In recent years, in addition to being an independent predictor of postoperative mortality and prognosis in gastrointestinal tumors, the potential application value of PNI in cardiovascular diseases has also been increasingly recognized (17, 19). Although some studies have shown that PNI can be used to predict short-term prognosis for certain heart surgeries, due to the urgency and complexity of AAAD disease, the predictive value of PNI in such cardiovascular emergencies is still unknown. Our study is also the first retrospective study on the predictive value of PNI for postoperative clinical adverse events in AAAD (38, 39).

Currently, the pathogenesis of aortic dissection (AD) is not clear. Many studies believe that the degradation of extracellular matrix in the middle layer of the aorta is closely related to the occurrence of AD (40). Activated lymphocytes can induce matrix metalloproteinase expression in smooth muscle cells of the aortic media, which is an important factor in promoting extracellular matrix degradation and plays an important role in the inflammatory response of AD, closely related to its prognosis (41, 42). Therefore, we believe that lymphocyte counts have potential value in predicting the prognosis of AD outcomes. At the same time, albumin, as an important indicator in clinical biochemistry testing, is usually used to evaluate the current nutritional status of the body, and can also indirectly reflect the degree of consumption of the body caused by disease, as most patients with inflammation-related diseases have acute or chronic consumption in their bodies (43). In this study, the PNI calculated by combining lymphocyte count and serum albumin was used to predict postoperative clinical adverse events. We believe that PNI has more accurate predictive value than its individual components. Currently, we have found that preoperative PNI < 42.45 is an independent risk factor for clinical adverse events after AAAD surgery. This finding can help clinicians make more appropriate clinical decisions in emergency situations of AAAD.

D-dimer is a non-specific fibrin degradation product that can reflect hyperfibrinolysis and hypercoagulable state in the human body. It is widely used for the diagnosis, efficacy evaluation, and prognosis prediction of thrombotic diseases (44, 45). During the occurrence of AD, due to the tearing and damage of the inner layer of the aorta, the coagulation system is rapidly activated, forming a false lumen thrombus, triggering a cascade reaction of coagulation, and activating the fibrinolysis system, leading to a rapid increase in the level of D-dimer in the serum. This has a good reference value in the diagnosis and differential diagnosis of AAAD (46). However, due to the fact that D-dimer is a highly sensitive but low-specificity detection indicator, its levels will significantly increase in cancer, infections, or any condition that may affect the body's coagulation function. Therefore, the reliability of using it as a prognostic indicator for AAAD still needs further verification. Research has also reported a correlation between the risk of in-hospital mortality in AAAD and the level of D-dimer (47). This may be due to the association between elevated D-dimer levels and serious complications such as postoperative acute renal failure, severe infection, and gastrointestinal bleeding. The level of elevated D-dimer can reflect the degree of disorder in the body's coagulation function to some extent. Although using D-dimer alone to predict the clinical outcome of AAAD has low specificity, our research results indicate that D-dimer > 15.05 μg/ml is an independent risk factor for postoperative clinical adverse events in AAAD, and has potential value in predicting the prognosis of AAAD. Therefore, we hope to increase its predictive reliability by combining it with other clinical indicators.

The results of this study indicate that preoperative PNI combined D-dimer are effective indicators for predicting postoperative clinical adverse events in patients with AAAD. Preoperative PNI < 42.45 and D-dimer > 15.05 μg/ml are independent risk factors for patients to experience postoperative clinical adverse events, which may provide more valuable predictive evaluation for the prognosis of patients with AAAD. This study is the first to apply PNI to the prediction of prognosis outcomes in cardiovascular emergencies such as AAAD. This composite inflammation-related indicator comprehensively considers the current nutritional status of the body, which can more reliably evaluate the prognosis outcomes. At the same time, we also found that the combination of PNI and the coagulation function indicator D-dimer had a significantly better predictive effect than using any single indicator alone (AUC = 0.843). The combination of the two indicators mentioned above for predicting the postoperative outcomes of AAAD patients has clear advantages, mainly because these clinical indicators do not increase the patient's medical costs or cause additional trauma, and are easy to obtain results in practical operations. This advantage also increases the clinical application value of these indicators.

The limitations of this study are as follows: (1) This is a single-center retrospective study, and the results may be limited by factors such as sample size, only representing the experience of this center in predicting adverse events of AAAD. In the future, more multi-center randomized controlled trials with larger sample sizes are needed to further verify the conclusions of this study; (2) This study only explored the relationship between preoperative PNI and D-dimer levels and postoperative adverse events, without further studying whether they have practical value in predicting the mid-to-long-term prognosis of AAAD, which is our next research direction.

## Conclusion

Preoperative PNI < 42.45 and D-dimer > 15.05 are independent predictive factors for adverse clinical events in patients with AAAD after surgery, and have potential application value for predicting postoperative prognosis. The combined use of these two indicators can further improve their predictive value.

## Data Availability

The raw data supporting the conclusions of this article will be made available by the authors, without undue reservation.

## References

[1] Nienaber CA Clough RE. Management of acute aortic dissection. Lancet. (2015) 385:800–11. 10.1016/S0140-6736(14)61005-9 25662791

[2] Rampoldi V Trimarchi S Eagle KA Nienaber CA Oh JK Bossone E Simple risk models to predict surgical mortality in acute type A aortic dissection: the international registry of acute aortic dissection score. Ann Thorac Surg. (2007) 83:55–61. 10.1016/j.athoracsur.2006.08.007 17184630

[3] Evangelista A Isselbacher EM Bossone E Gleason TG Eusanio MD Sechtem U Insights from the international registry of acute aortic dissection: a 20-year experience of collaborative clinical research. Circulation. (2018) 137:1846–60. 10.1161/CIRCULATIONAHA.117.031264 29685932

[4] Pacini D Di Marco L Fortuna D Belotti LM Gabbieri D Zussa C Acute aortic dissection: epidemiology and outcomes. Int J Cardiol. (2013) 167:2806–12. 10.1016/j.ijcard.2012.07.008 22882963

[5] Mészáros I Mórocz J Szlávi J Schmidt J Tornóci L Nagy L Epidemiology and clinicopathology of aortic dissection. Chest. (2000) 117:1271–8. 10.1378/chest.117.5.1271 10807810

[6] Trimarchi S Nienaber CA Rampoldi V Myrmel T Suzuki T Mehta RH Contemporary results of surgery in acute type A aortic dissection: the international registry of acute aortic dissection experience. J Thorac Cardiovasc Surg. (2005) 129:112–22. 10.1016/j.jtcvs.2004.09.005 15632832

[7] Pagni S Ganzel BL Trivedi JR Singh R Mascio CE Austin EH Early and midterm outcomes following surgery for acute type A aortic dissection. J Card Surg. (2013) 28:543–9. 10.1111/jocs.12170 23909254

[8] Shang W Ma M Ge YP Liu N Zhu JM Sun LZ. Analysis of risk factors of type a aortic dissection (TAAD) operation of frozen elephant trunk and total arch replacement. Eur Rev Med Pharmacol Sci. (2016) 20:4586–92. 27874962

[9] Halstead JC Spielvogel D Meier DM Rinke S Bodian C Malekan R Composite aortic root replacement in acute type A dissection: time to rethink the indications? Eur J Cardiothoracic Surg. (2005) 27:626–32. discussion 632–3. 10.1016/j.ejcts.2004.12.059 15784362

[10] Nagareddy P Smyth SS. Inflammation and thrombosis in cardiovascular disease. Curr Opin Hematol. (2013) 20:457–63. 10.1097/MOH.0b013e328364219d 23892572 PMC4086917

[11] Levi M van der Poll T. The role of natural anticoagulants in the pathogenesis and management of systemic activation of coagulation and inflammation in critically ill patients. Semin Thromb Hemost. (2008) 34:459–68. 10.1055/s-0028-1092876 18956286

[12] Liu Y Han L Li J Gong M Zhang H Guan X. Consumption coagulopathy in acute aortic dissection: principles of management. J Cardiothorac Surg. (2017) 12:50. 10.1186/s13019-017-0613-5 28606160 PMC5468986

[13] Guan XL Wang XL Liu YY Lan F Gong M Li HY Changes in the hemostatic system of patients with acute aortic dissection undergoing aortic arch surgery. Ann Thorac Surg. (2016) 101:945–51. 10.1016/j.athoracsur.2015.08.047 26603023

[14] Okina N Ohuchida M Takeuchi T Fujiyama T Satoh A Sakamoto T Utility of measuring C-reactive protein for prediction of in-hospital events in patients with acute aortic dissection. Heart Vessels. (2013) 28:330–5. 10.1007/s00380-012-0257-2 22570140

[15] Nogueiro J Santos-Sousa H Pereira A Devezas V Fernandes C Sousa F The impact of the prognostic nutritional index (PNI) in gastric cancer. Langenbecks Arch Surg. (2022) 407:2703–14. 10.1007/s00423-022-02627-0 35932298

[16] Onodera T Goseki N Kosaki G. Prognostic nutritional index in gastrointestinal surgery of malnourished cancer patients. Nihon Geka Gakkai Zasshi. (1984) 85:1001–5. 6438478

[17] Li H Cen K Sun W Feng B. Prognostic value of geriatric nutritional risk index in elderly patients with heart failure: a meta-analysis. Aging Clin Exp Res. (2021) 33:1477–86. 10.1007/s40520-020-01656-3 32766928

[18] Raposeiras Roubín S Abu Assi E Cespón Fernandez M Barreiro Pardal C Lizancos Castro A Parada JA Prevalence and prognostic significance of malnutrition in patients with acute coronary syndrome. J Am Coll Cardiol. (2020) 76:828–40. 10.1016/j.jacc.2020.06.058 32792081

[19] Cheng YL Sung SH Cheng HM Hsu PF Guo CY Yu WC Prognostic nutritional Index and the risk of mortality in patients with acute heart failure. J Am Heart Assoc. (2017) 6(6):e004876. 10.1161/JAHA.116.004876 28649089 PMC5669149

[20] Lin Y Chen Q Peng Y Chen Y Huang X Lin L Prognostic nutritional index predicts in-hospital mortality in patients with acute type A aortic dissection. Heart Lung. (2021) 50:159–64. 10.1016/j.hrtlng.2020.06.004 32690218

[21] Keskin HA Kurtul A Esenboğa K Çiçek MC Katırcıoğlu SF. Prognostic nutritional index predicts in-hospital mortality in patients with acute Stanford type A aortic dissection. Perfusion. (2021) 36:710–6. 10.1177/0267659120961937 33070761

[22] Nazerian P Mueller C Soeiro AM Leidel BA Salvadeo SAT Giachino F Diagnostic accuracy of the aortic dissection detection risk score plus D-dimer for acute aortic syndromes: the ADvISED prospective multicenter study. Circulation 137 (2018) 250–8. 10.1161/CIRCULATIONAHA.117.029457 29030346

[23] Clavien PA Barkun J de Oliveira ML Vauthey JN Dindo D Schulick RD The Clavien–Dindo classification of surgical complications: five-year experience. Ann Surg. (2009) 250:187–96. 10.1097/SLA.0b013e3181b13ca2 19638912

[24] Wu Q Li J Chen L Yan LL Qiu Z Shen Y Efficacy of interleukin-6 in combination with D-dimer in predicting early poor postoperative prognosis after acute Stanford type A aortic dissection. J Cardiothorac Surg. (2020) 15:172. 10.1186/s13019-020-01206-y 32677975 PMC7364558

[25] Gourd NM Nikitas N. Multiple organ dysfunction syndrome. J Intensive Care Med. (2020) 35:1564–75. 10.1177/0885066619871452 31455133

[26] Chen LW Dai XF Wu XJ Liao DS Hu YN Zhang H Ascending aorta and hemiarch replacement combined with modified triple-branched stent graft implantation for repair of acute DeBakey type I aortic dissection. Ann Thorac Surg. (2017) 103:595–601. 10.1016/j.athoracsur.2016.06.017 27553503

[27] Chen LW Wu XJ Dai XF Liao DS Li C Wang QM A self-adaptive triple-branched stent graft for arch repair during open type A dissection surgery. J Thorac Cardiovasc Surg. (2015) 149:1278–83.e1. 10.1016/j.jtcvs.2014.11.079 25598526

[28] Nienaber CA Powell JT. Management of acute aortic syndromes. Eur Heart J. (2012) 33:26–35b. 10.1093/eurheartj/ehr186 21810861

[29] Lu J Li P Ma K Li Y Yuan H Zhu J OPG/TRAIL ratio as a predictive biomarker of mortality in patients with type A acute aortic dissection. Nat Commun. (2021) 12:3401. 10.1038/s41467-021-23787-5 34099729 PMC8185077

[30] Tang Z Liu H Shao Y. Efficacy of CRP in combination with D-dimer in predicting adverse postoperative outcomes of patients with acute Stanford type A aortic dissection. J Cardiothorac Surg. (2022) 17:71. 10.1186/s13019-022-01818-6 35410359 PMC8996412

[31] Luo F Zhou XL Li JJ Hui RT. Inflammatory response is associated with aortic dissection. Ageing Res Rev. (2009) 8:31–5. 10.1016/j.arr.2008.08.001 18789403

[32] Kalkan ME Kalkan AK Gündeş A Yanartaş M Oztürk S Gurbuz AS Neutrophil to lymphocyte ratio: a novel marker for predicting hospital mortality of patients with acute type A aortic dissection. Perfusion. (2017) 32:321–7. 10.1177/0267659115590625 26467992

[33] Zhou Q Chai XP Fang ZF Hu XQ Tang L. Association of plasma pentraxin-3 levels on admission with in-hospital mortality in patients with acute type A aortic dissection. Chin Med J. (2016) 129:2589–95. 10.4103/0366-6999.192785 27779166 PMC5125338

[34] Zeng T Shi L Ji Q Shi Y Huang Y Liu Y Cytokines in aortic dissection. Clin Chim Acta. (2018) 486:177–82. 10.1016/j.cca.2018.08.005 30086263

[35] Hsieh WC Henry BM Hsieh CC Maruna P Omara M Lindner J. Prognostic role of admission C-reactive protein level as a predictor of in-hospital mortality in type-A acute aortic dissection: a meta-analysis. Vasc Endovascular Surg. (2019) 53:547–57. 10.1177/1538574419858161 31248351

[36] Ruparelia N Chai JT Fisher EA Choudhury RP. Inflammatory processes in cardiovascular disease: a route to targeted therapies. Nat Rev Cardiol. (2017) 14:133–44. 10.1038/nrcardio.2016.185 27905474 PMC5525550

[37] Casas R Castro-Barquero S Estruch R Sacanella E. Nutrition and cardiovascular health. Int J Mol Sci. (2018) 19(12):3988. 10.3390/ijms19123988 30544955 PMC6320919

[38] Wada H Dohi T Miyauchi K Jun S Endo H Doi S Relationship between the prognostic nutritional index and long-term clinical outcomes in patients with stable coronary artery disease. J Cardiol. (2018) 72:155–61. 10.1016/j.jjcc.2018.01.012 29496336

[39] Lee SI Ko KP Choi CH Park CH Park KY Son KH. Does the prognostic nutritional index have a predictive role in the outcomes of adult cardiac surgery? J Thorac Cardiovasc Surg. (2020) 160:145–153.e3. 10.1016/j.jtcvs.2019.08.069 31627943

[40] Wu X Ye J Cai W Yang X Zou Q Lin J LDHA mediated degradation of extracellular matrix is a potential target for the treatment of aortic dissection. Pharmacol Res. (2022) 176:106051. 10.1016/j.phrs.2021.106051 34973467

[41] Oviedo-Orta E Bermudez-Fajardo A Karanam S Benbow U Newby AC. Comparison of MMP-2 and MMP-9 secretion from T helper 0, 1 and 2 lymphocytes alone and in coculture with macrophages. Immunology. (2008) 124:42–50. 10.1111/j.1365-2567.2007.02728.x 17949416 PMC2434380

[42] Schönbeck U Mach F Sukhova GK Murphy C Bonnefoy JY Fabunmi RP Regulation of matrix metalloproteinase expression in human vascular smooth muscle cells by T lymphocytes: a role for CD40 signaling in plaque rupture? Circ Res. (1997) 81:448–54. 10.1161/01.RES.81.3.448 9285647

[43] Ohsuzu F. The roles of cytokines, inflammation and immunity in vascular diseases. J Atheroscler Thromb. (2004) 11:313–21. 10.5551/jat.11.313 15644584

[44] Weitz JI Fredenburgh JC Eikelboom JW. A test in context: D-dimer. J Am Coll Cardiol. (2017) 70:2411–20. 10.1016/j.jacc.2017.09.024 29096812

[45] Favresse J Lippi G Roy PM Chatelain B Jacqmin H Ten Cate H D-dimer: preanalytical, analytical, postanalytical variables, and clinical applications. Crit Rev Clin Lab Sci. (2018) 55:548–77. 10.1080/10408363.2018.1529734 30694079

[46] Sodeck G Domanovits H Schillinger M Ehrlich MP Endler G Herkner H D-dimer in ruling out acute aortic dissection: a systematic review and prospective cohort study. Eur Heart J. (2007) 28:3067–75. 10.1093/eurheartj/ehm484 17986466

[47] Halaby R Popma CJ Cohen A Chi G Zacarkim MR Romero G D-dimer elevation and adverse outcomes. J Thromb Thrombolysis. (2015) 39:55–9. 10.1007/s11239-014-1101-6 25006010 PMC4300425

